# Adipose Mesenchymal Stem Cell-Derived Exosomes Enhance PC12 Cell Function through the Activation of the PI3K/AKT Pathway

**DOI:** 10.1155/2021/2229477

**Published:** 2021-10-15

**Authors:** Yong Xie, Yangping Chen, Yuyuan Zhu, Xia Chen, Tiecheng Lin, Dan Zhou

**Affiliations:** ^1^Department of Obstetrics and Gynecology, The First People's Hospital of Foshan, Foshan 528000, China; ^2^Department of Galactophore Surgery, The First People's Hospital of Foshan, Foshan 528000, China

## Abstract

Transplantation of mesenchymal stem cells has been considered as an auspicious treatment for repairing nerve injuries. The rat adrenal pheochromocytoma cell line (PC12) is one of the traditional models for the study of neuronal differentiation and neuroregeneration in vitro. However, the effects of adipose mesenchymal stem cell-derived exosomes (ADSC-exo) on PC12 cells remain unclear and to be elucidated. In our study, the effects of ADSC-exo on PC12 cells were investigated. ADSC-exo were isolated by ultracentrifugation and characterized by transmission electron microscopy, flow nanoanalysis, and western blot. The effects of ADSC-exo on PC12 cell proliferation, migration, apoptosis, and the protein levels were analyzed using CCK-8 assay and EdU incorporation assay, transwell migration assay and scratch wound assay, flow cytometry, and western blot, respectively. We successfully isolated and purified exosomes from ADSC supernatant and found that ADSC-exo treatment significantly promoted PC12 cell proliferation and migration, inhibited their apoptosis, and activated the PI3K/AKT pathway, while PI3K/AKT signaling repression using LY294002 exhibited the opposite effects. The results showed that ADSC-exo promoted proliferation and migration and inhibited apoptosis of PC12 through the activation of the PI3K/AKT pathway. Thus, the effect of ADSC-exo on PC12 cells may suggest ADSC-exo may be a promising therapeutic for nerve damage.

## 1. Introduction

Radical pelvic surgery, such as that performed for cervical cancer and ovarian cancer, often leads to dysfunction in urination. This may be related to pelvic plexus nerve injury caused by surgery [1–3]. Because of the limited capacity of nerve tissue for self-repair, there is no effective treatment at present. Recent studies have confirmed that mesenchymal stem cells (MSCs) can promote repair of nerve injury and improvement of function [4–7]. Adipose mesenchymal stem cells (ADSCs) are one kind of MSCs derived from adipose tissue, and they have the capacity for self-renewal and differentiation into adipocytes, osteoblasts, chondrocytes, and nerve cells [8]. A growing body of research has shown that the therapeutic effect of ADSCs may not depend on its multidirectional differentiation and is more likely to be related to the cytokines or exosomes secreted by cells [9–11].

Exosomes are membranous vesicles secreted by almost all active cells with a diameter of 30~120 nm that contain bioactive substances such as protein, lipid, and nucleic acids (DNA, microRNAs, lncRNA, circRNA, and other noncoding RNAs), which are easy to fuse with the cell membrane of neighboring cells, and selectively inject bioactive substances into target cells, thus realizing intercell signal transmission [12–14]. Some researchers have found that adipose mesenchymal stem cell exosomes (ADSC-exo) have similar functions as ADSC, which can promote repair of heart muscle, kidney, liver, and other tissues after the damage occurred [8, 15, 16].

Rat pheochromocytoma cell line (PC12) is a commonly used cell model in laboratory. It has the general characteristics of nerve cells and has been widely used in the research of neurophysiology and neuropharmacology [17, 18]. In this study, we aimed to determine whether ADSC-exo have beneficial action in the treatment of PC12 cells in vitro and to further verify that the mechanism of which may involve the PI3K/AKT signaling pathway.

## 2. Results

### 2.1. Identification of ADSCs

**Figure 1:**
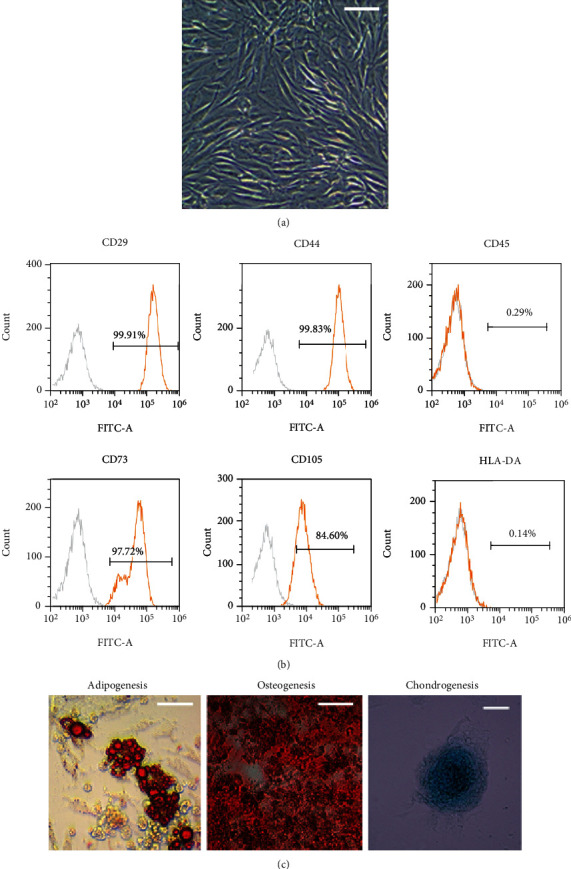
Isolation and characterization of ADSCs. (a) Morphological characteristics of ADSCs. Scale bar = 100 *μ*m. (b) Adipogenic, osteogenic, and chondrogenic differentiation of ADSCs. Adipogenesis was positively determined by oil red O staining, osteogenesis was positively determined by alizarin red staining, and chondrogenesis was positively determined by alcian blue staining. Scale bar = 100 *μ*m. (c) Flow cytometry analysis of ADSC surface markers. CD29, CD44, CD73, and CD105 were highly expressed on ADSCs, whereas CD45 and HLA-DR were rarely expressed. ADSCs: adipose-derived mesenchymal stem cells.

The primary ADSCs adhered to the wall and grew in a spindle shape. After passage, the cells grew in a parallel arrangement or swirl morphology (Figure 1(a)). Flow cytometry demonstrated that ADSCs were highly expressed mesenchymal stem cell surface markers CD29, CD44, CD73, and CD105, while for the lowly expressed CD45 and HLA-DR (Figure 1(b)). After 14 days of osteogenic induction of ADSCs, calcium nodules appeared and stained bright red by alizarin red staining. After induction with adipogenic medium, the lipid droplets gradually became more and larger after 21 d, which were stained bright red by oil red O staining. After induction of chondrogenic differentiation, proteoglycans were deposited and stained blue by alcian blue staining (Figure 1(c)).

### 2.2. Identification of ADSC-exo

**Figure 2:**
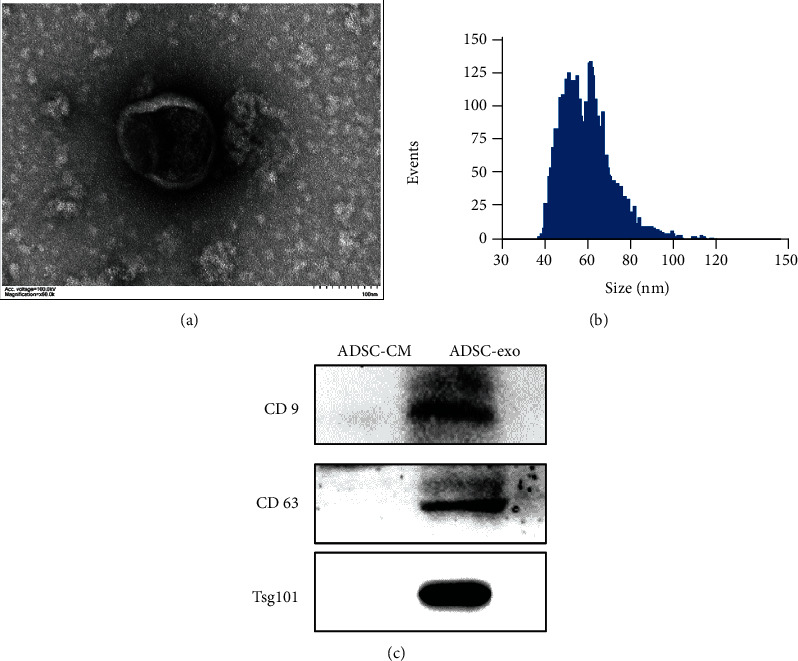
Isolation and characterization of ADSC-exo. (a) TEM photomicrographs of exosomes. Scale bar = 100 nm. (b) Nanotrack analysis size distribution of exosomes by flow nanoanalysis. (c) The expression of exosomal marker proteins CD9, CD63, and TSG101 by western blot assay. ADSC-exo: exosomes derived from adipose mesenchymal stem cells; TEM: transmission electron microscopy; ADSC-CM: conditioned medium of adipose mesenchymal stem cells.

The ADSC-exo showed typical tea-saucer type morphology with the diameters in size from 30 to 120 nm (Figure 2(a)) under a transmission electron microscope. The concentration of ADSC-exo was 5.43 × 10^10^ particles/ml, with an average particle size of 74.53 nm, as detected by Flow NanoAnalyzer (Figure 2(b)). As demonstrated in western blotting, exosomal surface markers (CD63, CD9, and TSG101) were positively expressed in the ADSC-exo as expected (Figure 2(c)).

### 2.3. ADSC-exo Promote PC12 Cell Proliferation

**Figure 3:**
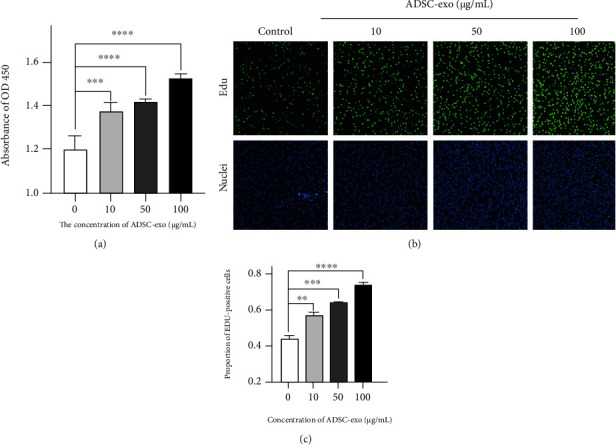
ADSC-exo promote proliferation of PC12 cells. (a) PC12 cells were incubated with various concentrations of ADSC-exo (0, 10, 50, and 100 *μ*g/ml) for 48 h, and cell proliferation was determined by CCK-8 assay. (b, c) PC12 cells were treated with various concentrations of ADSC-exo (0, 10, 50, and 100 *μ*g/ml) for 24 h, and then, the cells were incubated with EdU for 3 h before fixation and stained. EdU-positive cells are expressed as percentages of positively stained cells compared with total stained cells per field. ADSC-exo: exosomes derived from adipose mesenchymal stem cells; CCK-8: Cell Counting Kit-8; EdU: 5-ethynyl-20-deoxyuridine. ^∗^*P* < 0.05; ^∗∗^*P* < 0.01; ^∗∗∗^*P* < 0.001; ^∗∗∗∗^*P* < 0.0001.

CCK-8 analysis (Figure 3(a)) and EdU analysis (Figures 3(b) and 3(c)) for cell proliferation showed that ADSC-exo significantly promoted PC12 cell proliferation in an exosome dose-dependent manner.

### 2.4. ADSC-exo Promote PC12 Cell Migration

**Figure 4:**
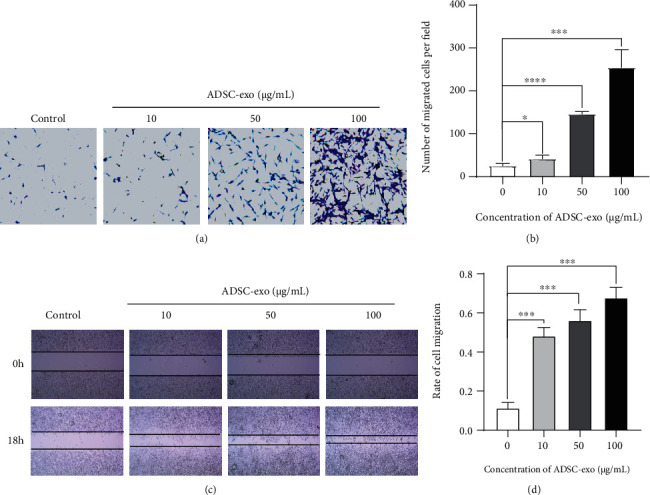
ADSC-exo promote migration of PC12 cells. (a, b) Transwell migration assay for the migratory ability of PC12 cells with various concentrations of ADSC-exo (0, 10, 50, and 100 *μ*g/ml) treatment for 24 h. (c, d) Scratch wound assay for the migratory ability of PC12 cells with various concentrations of ADSC-exo (0, 10, 50, and 100 *μ*g/ml) treatment for 18 h. ADSC-exo: exosomes derived from adipose mesenchymal stem cells; ADSC-exo: exosomes derived from adipose mesenchymal stem cells. ^∗^*P* < 0.05; ^∗∗^*P* < 0.01; ^∗∗∗^*P* < 0.001; ^∗∗∗∗^*P* < 0.0001.

The results of transwell assay (Figures 4(a) and 4(b)) and scratch wound assay (Figures 4(c) and 4(d)) displayed that the migratory ability of PC12 cells cultured with ADSC-exo was enhanced in an exosome dose-dependent manner.

### 2.5. ADSC-exo Inhibit PC12 Cell Apoptosis

**Figure 5:**
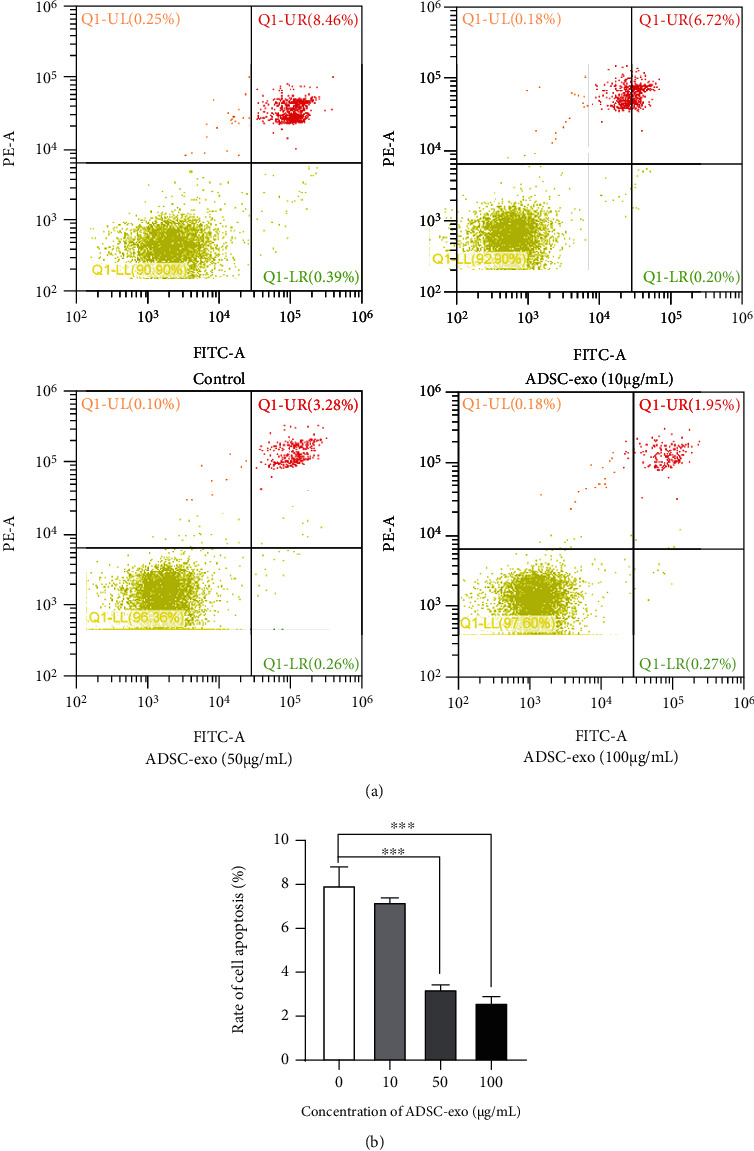
ADSC-exo inhibit apoptosis of PC12 cells. (a) The PC12 cell mechanical injury model was established by the scratch method to induce cell apoptosis; then, cell apoptosis of PC12 cells treated with various concentrations of ADSC-exo (0, 10, 50, and 100 *μ*g/ml) was monitored by flow cytometry assay. (b) Percentage of total apoptotic cells. ADSC-exo: exosomes derived from adipose mesenchymal stem cells. ^∗^*P* < 0.05; ^∗∗^*P* < 0.01; ^∗∗∗^*P* < 0.001; ^∗∗∗∗^*P* < 0.0001.

ADSC-exo treatment effectively relieved the negative effect of mechanical injury on PC12 cells in an exosome dose-dependent manner. The mechanical injury-mediated increase of apoptosis rate was sharply reduced with the treatment of ADSC-exo by flow cytometry (Figures 5(a) and 5(b)).

### 2.6. Regulatory Function of PI3K/AKT Signaling in PC12 Cell Proliferation, Migration, and Antiapoptosis

**Figure 6:**
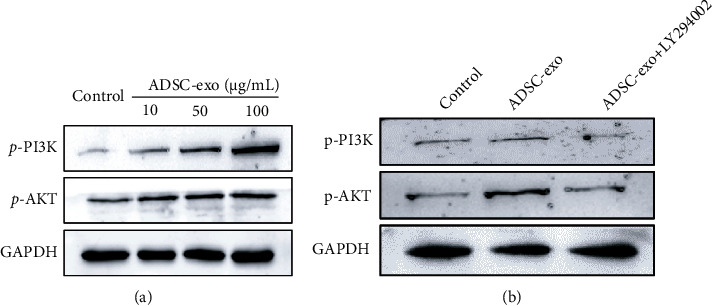
Western blot analysis for the expression levels of PI3K/AKT in PC12 cells. (a) The expression levels of *p*-PI3K and *p*-AKT were analyzed in PC12 cells with various concentrations of ADSC-exo (0, 10, 50, and 100 *μ*g/ml) treatment. (b) The ADSC-exo-induced levels of *p*-PI3K and *p*-AKT were interrupted by LY294002 (10 *μ*M). ADSC-exo: exosomes derived from adipose mesenchymal stem cells.

**Figure 7:**
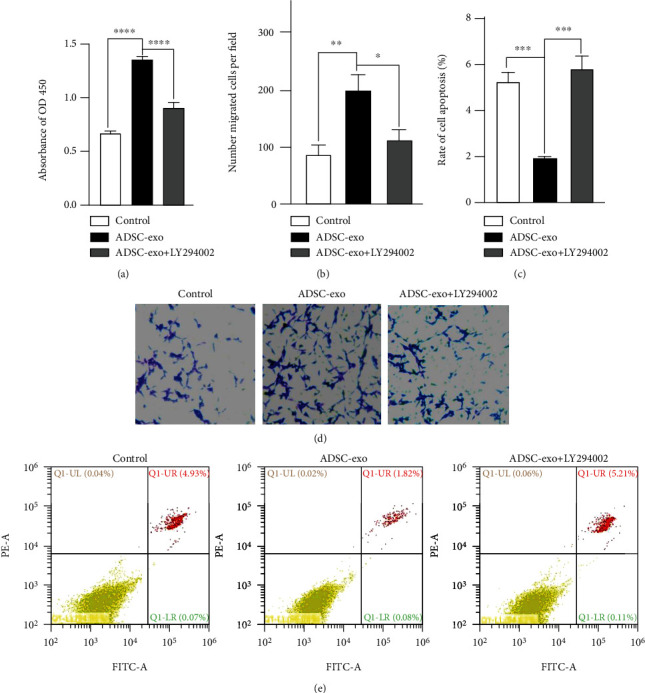
Inhibition of the PI3K/AKT signaling pathway could influence the biological behaviors of PC12 cells. (a) CCK-8 assay. The addition of LY294002 could inhibit the proliferation of PC12 cells treated with ADSC-exo (100 *μ*g/ml). (b, d) Transwell migration assay. LY294002 could decrease the cell migration promoted by ADSC-exo (100 *μ*g/ml) in PC12 cells. (c, e) Flow cytometry assay. The antiapoptotic effects of ADSC-exo (100 *μ*g/ml) were reversed after suppressing the Wnt/*β*-catenin signaling pathway by LY294002. ADSC-exo: exosomes derived from adipose mesenchymal stem cells; CCK-8: Cell Counting Kit-8. ^∗^*P* < 0.05; ^∗∗^*P* < 0.01; ^∗∗∗^*P* < 0.001; ^∗∗∗∗^*P* < 0.0001.

As one of the important intracellular signaling pathways, the PI3K/Akt pathway plays a key role in inhibiting apoptosis and promoting proliferation and migration. The results of western blotting showed that ADSC-exo significantly increase protein levels of *p*-PI3K and *p*-AKT in PC12 cells in a dose-dependent manner (Figure 6(a)), suggesting that regulation of ADSC-exo-dependent proliferation, migration, and antiapoptosis may occur through the PI3K/AKT signaling pathway. To further confirm that the activation of PI3K/Akt is responsible for the progression of PC12 cells by ADSC-exo treatment, PC12 cells were treated with ADSC-exo in the presence or absence of the PI3K inhibitor LY294002 (Figure 6(b)). Our western blot results suggested that the ADSC-exo-mediated activation of PI3K/Akt signaling could be blocked by LY294002 (Figure 6(b)). The CCK-8 assay and transwell assay displayed that ADSC1-exo-induced cell proliferation (Figure 7(a)) and migration (Figures 7(b) and 7(d)) were significantly inhibited by LY294002. Flow cytometry results showed that the enhanced antiapoptosis (Figures 7(c) and 7(e)) of ADSC-exo-treated PC12 cells was also inhibited by LY294002.

## 3. Discussion

Radical pelvic surgery can improve the survival rate of patients, but it can also damage the pelvic nerves [3, 21]. Because of the limited capacity of peripheral nerves for self-repair, it is difficult to achieve complete functional recovery of nerve after injury, especially for injury to the long nerves and nerve deficiency injury [22]. A large number of experiments have confirmed that MSCs have therapeutic effects on nerve injury [23], but the specific mechanism remains unclear [24]. With the further development of studies, more and more researchers have gradually realized that the therapeutic effect of MSCs is closely related to the cytokines or other active substances secreted by cells, such as exosomes [25, 26]. MSC-exo is a vesicular structure secreted by MSCs that contains a variety of genetic materials and protein signals and plays an important role in intercellular signaling [27, 28].

Stem-cell-derived exosomes have been widely used in various pathological diagnoses and treatments, such as those for neurodegenerative diseases, wound repair, antiaging, and tumor suppression. They are expected to become a new method for prognostic analysis and monitoring [29]. Unlike stem cells, MSC-exo has little immunogenicity and no tumorigenicity, which allows further implementation of cell-free therapy strategy [30, 31]. The ability of MSC-exo to promote regeneration and repair is closely related to the function of the original cells, and the basic characteristics of original cells and the difficulty of isolation and acquisition determine the prospects of MSC-exo for use [32, 33]. ADSCs are more abundant and convenient to use, and ADSC-exo can promote the repair of damaged tissues, similar to the way ADSCs do [8, 29, 34].

One study on stress urinary incontinence found the density of rhabdoid muscle fibers and peripheral nerve fibers in the urethras of rats treated with ADSC-exo to be greater than that of the untreated group [35]. Yin et al. [36] showed that ADSC-exo promoted the repair of sciatic nerve injury by reducing apoptosis and autophagy of Schwann cells in the sciatic nerve injury model. According to research findings, pigment epithelium derivative factor modified ADSC-exo activated autophagy and inhibited apoptosis in neurons, thereby alleviating cerebral ischemia-reperfusion injury [37]. Recently, it has been shown that ADSC-exo can deliver miR-30d-5p to inhibit microglial autophagy, ultimately promoting microglial polarization to an anti-inflammatory phenotype and reversing neuronal damage [38].

In this experiment, we successfully isolated ADSCs from adipose tissues and extracted ADSC-exo from the cell supernatant of ADSCs. Under a transmission electron microscope, ADSC-exo showed a typical vesicular structure. At the same time, our results confirmed that ADSC-exo could significantly promote cell proliferation and migration and reduce cell apoptosis of PC12 by activating the PI3K/Akt signaling pathway.

Above all, our results suggested the positive role of ADSC-exo through PI3K/Akt signaling in repair of nerve injury. However, there are still some limitations to our study. More studies should be carried out in the aspect of the association between the PI3K/Akt signaling pathway, ADSC-exo, and repair of nerve injury, and we would like to further investigate it in our future research.

## 4. Materials and Methods

### 4.1. Cell Line and Culture

PC12 cells were purchased from Procell Life Science and Technology Co., Ltd. (Wuhan, China) and cultured in RPMI-1640 medium containing 10% FBS (ExCell Bio, China) and 1% penicillin-streptomycin (Gibco, USA).

### 4.2. Isolation and Identification of ADSCs

Subcutaneous adipose tissue was obtained from the women who delivered by cesarean section, and the samples were obtained with the approval of the Ethics Committee of Foshan First People's Hospital and the informed consent of donors.

The methods for isolation and culture of ADSCs were described in previously published papers [19, 20]. Briefly, adipose tissue was digested with collagenase type I for 2-4 h at 37°C and the supernatant was removed after centrifugation at 1000 r/min for 5 min. Cells were suspended in MEM ALPHA (Gibco, USA) containing 10% FBS (ExCell Bio, China) and 1% penicillin-streptomycin (Gibco, USA). Cells were passaged until reaching approximately 80%-90% confluence.

Surface markers of MSCs were detected for the third generation of ADSCs by flow cytometry. ADSCs were incubated for half an hour with anti-human-CD29-PE, anti-human-CD44-FITC, anti-human-CD45-FITC, anti-human-CD73-PE, anti-human-CD105-FITC, and anti-human-HLA-DR-FITC monoclonal antibodies (BioLegend, USA) and acquired on CytoFLEX Flow Cytometer (Beckman Coulter, USA). FITC and PE IgG Isotype Control were used for differentiation of positive and negative signals.

The adipogenic induction medium (Cyanogen, China), osteogenic induction medium (Cyanogen, China), and chondrogenic induction medium (Cyanogen, China) were used for the third generation of ADSCs. After 14-21 days of induction, lipid droplets were identified by oil red staining, calcium nodules were identified by alizarin red staining, and proteoglycans were identified by alizarin red, respectively.

### 4.3. Exosome Isolation and Analysis

ADSCs at passages 3-10 were cultured in an exosome-clear medium, and cell supernatant was collected 48 h later. ADSC-exo was isolated and purified from the ADSC supernatant by ultracentrifugation and Exojucie™ exosome purification and concentration kit (Weina Biomedicine, China) according to the manufacturer's instructions. The morphology of exosomes was observed by transmission electron microscopy. Briefly, the ADSC-exo was fixed in 2% acetate and spotted onto a copper grid, then stained with uranium dioxide acetate, and dried at room temperature for several minutes. The samples were analyzed at 100 kV. The particle diameter and concentration were analyzed by flow nanoanalysis using nanoflow cytometry (NanoFCM Co. Ltd., China) following the manufacturer's instructions. Then, the exosomal markers (CD63, CD9, and TSG101) were detected by western blotting. ADSC-exo and PC12 cells were cocultured at a concentration of 10, 50, and 100 *μ*g/ml, respectively.

### 4.4. Cell Proliferation Assay

Cell proliferation was determined by Cell Counting Kit-8 (CCK-8) assay (APExBIO, USA) and 5-ethynyl-20-deoxyuridine (EdU) incorporation assay (Beyotime, China). Briefly, PC12 cells were plated in 96-well plates at a density of 5 × 10^3^ cells per well with 100 *μ*l of complete culture medium. After 24 h, cells were treated with the indicated amount of ADSC-exo, as previously described. After continuous culture for 2 days, 10 *μ*l CCK-8 solution was added to each well and incubated for 1 h at 37°C. The absorbance value at 450 nm was detected by a microplate reader (Thermo MK3, USA).

For the EdU incorporation assay, PC12 cells were seeded in 6-well plates at a rate of 5 × 10^5^ cells/well. After 24 h, cells were treated with the indicated amount of ADSC-exo, as previously described. After continuous culture for 24 h, cells were treated with EdU solution (Beyotime, China) for 2 h and incubated in click reaction solution for 30 min in the dark at 37°C. Cells were stained according to the manufacturer's instructions, then imaged under a fluorescence microscope, and the percentage of EDU-positive cells was calculated.

### 4.5. Cell Migration Assay

Cell migration was determined by scratch wound assay and transwell migration assay. Briefly, the serum-free cell suspension (5 × 10^4^ PC12 cells in 100 *μ*l volume per well) was placed on the upper layer of transwell, and the medium with the indicated amount of ADSC-exo was added to the bottom chambers, as previously described. After 24 h, we used a cotton swab to wipe off the cell membrane surface, and crystal violet was used for staining. Observe the cells and count the stained migrated cells in the lower chamber.

For the scratch wound assay, a culture insert (Ibidi, Martinsried, Germany) was placed in the middle of 24-well culture plates. Subsequently, PC12 cells were seeded at a density of 5 × 10^5^/ml (70 *μ*l volume) per well. After 24 h, the culture insert was carefully removed, and PC12 cells were treated with the indicated amount of ADSC-exo for 18 h, as previously described. The area of cell migration was imaged by an inverted microscope (Olympus IX 71, 100x magnification, Olympus, Japan) and calculated by ImageJ software v1.8 (National Institutes of Health, USA).

### 4.6. Cell Apoptosis Analysis

The PC12 cell mechanical injury model was established by the scratch method to induce cell apoptosis; then, cells were treated with the indicated amount of ADSC-exo, as previously described. An Annexin V-fluorescein isothiocyanate (FITC)/propidium iodide (PI) kit (BD Biosciences, USA) was used according to the manufacturer's instruction. Briefly, Annexin V-FITC (5 *μ*l) and PI (5 *μ*l) solution were added into 100 *μ*l cell suspension, respectively. Following, the cells were incubated at room temperature for 15 min in the dark. PC12 cells were detected and analyzed by flow cytometry (Beckman CytoFLEX, USA).

### 4.7. Western Blot Analysis

The concentration of proteins was measured by the BCA kit, and 30 *μ*g of total proteins was loaded on to 10% SDS-PAGE and then transferred to PVDF membranes (Millipore, USA). The membranes were blocked with 5% skimmed milk for 2 h and then incubated with primary antibodies (dilution ratio, 1 : 1 000) at 4°C overnight. After washing 3 times with TBST, the corresponding horseradish peroxidase-labeled secondary antibodies (dilution ratio, 1 : 5 000) were added to incubation for 1 h. Electrochemiluminescence reagent (Tanon, China) was dropped into the membranes after washing 3 times with TBST. Finally, the protein expression was observed by chemiluminescence gel imaging analysis instrument (Tanon, China). Monoclonal antibodies for TSG101 and CD63 were from Santa Cruz Biotechnology (Santa Cruz, USA), monoclonal antibodies for CD9 were from Affinity Biosciences (Affinity, USA), and monoclonal antibodies for phospho-AKT, phospho-PI3K, and GAPDH were from Bioss Biotechnology (Bioss, China).

### 4.8. Statistical Analysis

Data are expressed as mean ± standard deviation. The unpaired *t*-test was used to explain the differences between the groups. *P* < 0.05 was considered statistically significant. All experimental data were analyzed and visualized using GraphPad Prism 8.0.

## Data Availability

The datasets used and/or analyzed during the current study are available from the corresponding author upon reasonable request.

## Abbreviations

ADSC-exo: Adipose mesenchymal stem cell-derived exosomes
MSCs: Mesenchymal stem cells
ADSCs: Adipose mesenchymal stem cells
PC12: Mouse pheochromocytoma cell line
CCK-8: Cell Counting Kit-8
EdU: 5-Ethynyl-20-deoxyuridine
FITC: Fluorescein isothiocyanate
PI: Propidium iodide
ADSC-CM: Conditioned medium of adipose mesenchymal stem cells.
